# From high- to one-dimensional dynamics of decision making: testing simplifications in attractor models

**DOI:** 10.1007/s10339-020-00953-z

**Published:** 2020-02-03

**Authors:** Martin Schoemann, Stefan Scherbaum

**Affiliations:** 1 Department of Psychology, Technische Universität Dresden, Zellescher Weg 17, 01069 Dresden, Germany; 2 Department of Management/MAPP, Aarhus University, Fuglesangs Allé 4, 8210 Aarhus V, Denmark

**Keywords:** Attractor model, Decision making, Process dynamics, Sequence effects, Choice history

## Abstract

Computational models introduce simplifications that need to be understood and validated. For attractor models of decision making, the main simplification is the high-level representation of different sub-processes of the complex decision system in one dynamic description of the overall process dynamics. This simplification implies that the overall process dynamics of the decision system are independent from specific values handled in different sub-processes. Here, we test the validity of this simplification empirically by investigating choice perseveration in a nonverbal, value-based decision task. Specifically, we tested whether choice perseveration occurred irrespectively of the attribute dimension as suggested by a simulation of the computational model. We find evidence supporting the validity of the simplification. We conclude that the simplification might capture mechanistic aspects of decision-making processes, and that the summation of the overall process dynamics of decision systems into one single variable is a valid approach in computational modeling. Supplement materials such as empirical data, analysis scripts, and the computational model are publicly available at the Open Science Framework (osf.io/7fb5q).

## Introduction

Computational modeling is an integral part of many areas in cognitive science since it offers a deeper understanding of the mechanical underpinnings of the processes of interest, such as decision making. An essential part of this understanding is to comprehend the implications of simplification, which is inherent to all models (McClelland 2009). A typical simplification is to aggregate complex stimuli into one input value to the model, that is, combining multiple attributes of a choice-option in decision making. Recently, this important simplification has been applied for the modeling of decision processes in delay discounting, which denotes the devaluation of an option’s value (usually money) by the delay with which it becomes available (Fredericket al. 2002; Malkoc and Zauberman 2019). Though such decisions are inherently multi-attributive (time and value of the available options have to be weighted against each other), they have been modeled using models that accumulate a single evidence measure for the options available, be it simple sequential sampling models (Dai and Busemeyer 2014; Dai et al. 2018; Zhao et al. 2019) for the decision process within a trial, or more complex attractor models (Scherbaum et al. 2016; Senftleben et al. 2019b) for the decision process within and across trials. However, one might question, whether this simplification is valid (Amasino et al. 2019; Cheng and González-Vallejo 2016; Dai et al. 2018), especially when modeling complex decision patterns across trials with attractor models. Here, we test the validity of this assumption for the predictions from an attractor model.

**Fig. 1:**
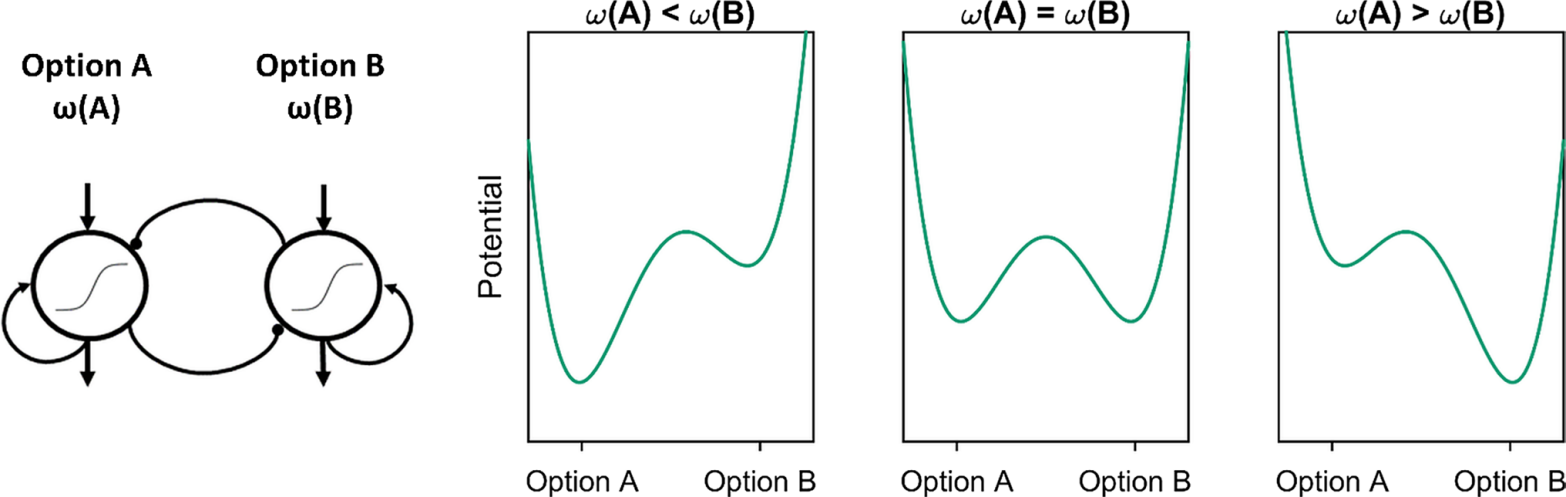
Possible state spaces for a simple neural attractor model for the choice between option A and option B given different settings for the subjective value [*ω*(*A*) and *ω*(*B*)] of both options. If the combined values of the options differ (left and right panel), the attractor of the high value option is deeper and thus makes it more likely that the system settles in the high value decision state. If both options have equal combined values (middle panel), both attractors are equally deep and there is no inherently more likely decision state

In attractor models of decision making (Scherbaum et al. 2008; Usher and McClelland, 2001; van Rooij et al. 2013), options are represented as self-sustainable active patterns of activation that constitute attractors in the system’s state space (Miller 2016; Rolls 2010; Wang 2008). In such models, the stability of each attractor is determined by the combined value of the respective option (see Fig. 1; cf. Scherbaum et al. 2008). The combined values, in turn, are given by a function ω combining the features of each option into one overall-value (Farashahi et al. 2019). For example, in delay discounting, one could combine the time of delivery and the value of an option by discounting the value by the time according to an individual discounting factor (Doyle 2013; Green and Myerson 2004; Green et al. 2005). The combined result would be the subjective value of the respective option. By simplifying the derivation and representation of the combined value, the attractor model is inherently independent from specific values on the feature/attribute level and only captures the continuous competition of the options on the subjective value level.

However, it is unclear whether this simplification is valid. The general plausibility of attractor models of decision making is supported by a series of studies linking perceptual and value-based decision making with activity in competitive neural networks located in the prefrontal cortex (Bogacz et al. 2011; Hunt et al. 2012; Jocham et al. 2012; Usher and McClelland 2001; Wang 2008, 2012; Wong et al. 2007). Additionally, computational modeling of competitive attractor networks and noninvasive brain stimulation has recently been used to decompose the connection between neural and choice variability (Bonaiuto et al. 2016; Hämmerer et al. 2016). Yet, a test of the validity of the simplification is still outstanding. To perform this test, we chose a case in which attractor models make specific predictions across trials of decisions: choice perseveration.

**Fig. 2:**
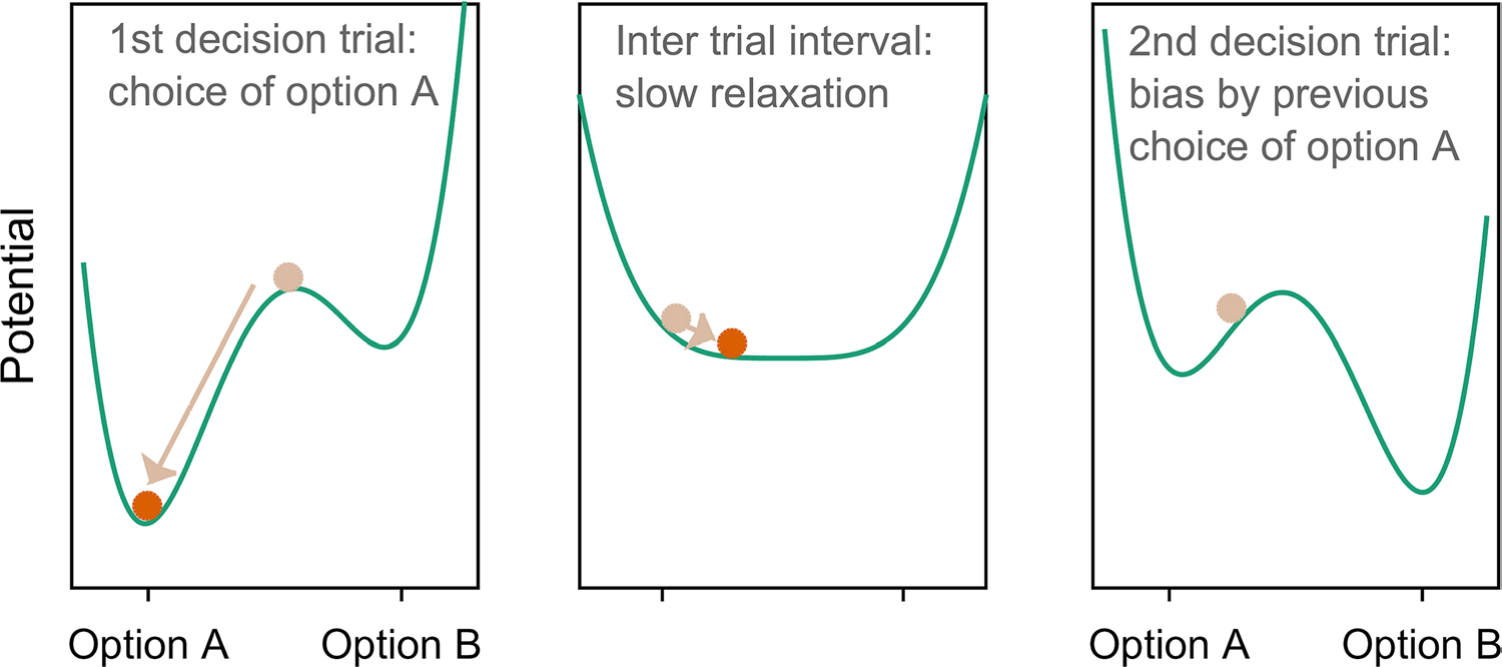
Inter-trial dynamics according to the attractor model. After choosing option A in a first trial, the system’s inertia leads to a slow relaxation to the neutral start point during the inter-trial interval; when the second decision trial starts, the system has not yet settled in the neutral start point and is still in vicinity of the attractor of option A, thus creating a bias toward option A

Due to inertia and residual activity, choice perseveration naturally emerges from neural-inspired dynamic systems such as attractor models (see Fig. 2; Alós-Ferrer et al. 2016; Gao et al. 2009; Hämmerer et al. 2016; Scherbaum et al. 2008; Townsend and Busemeyer 1989).

**Fig. 3:**
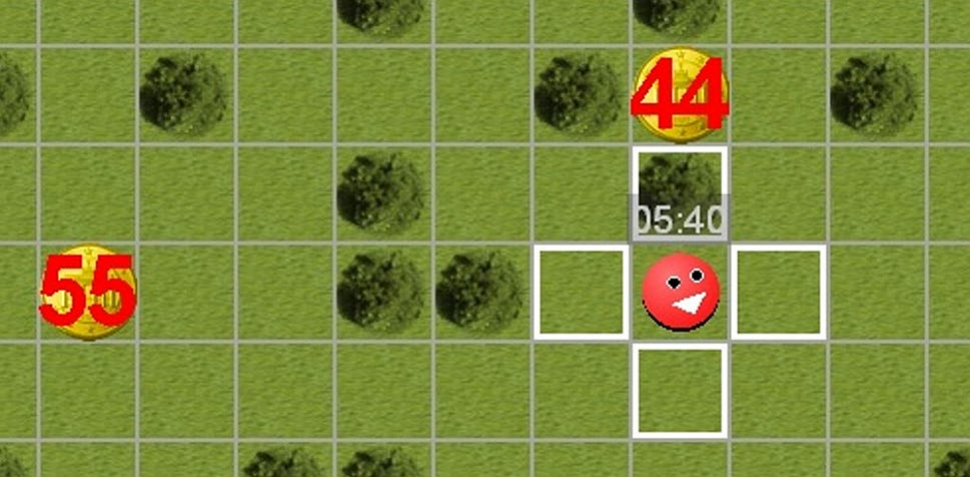
Display detail from the nonverbal computer game. Note: Participants control the red avatar by clicking into one of four horizontally or vertically adjacent fields outlined in white. In each trial, participants choose between two coins by moving the avatar field-by-field until they reach the chosen coin. Participants can move freely; trees (in dark green) are included for better spatial orientation, they do not restrict movement. The time within each block is displayed above the avatar. Accumulated credits (1 credit = 1/10 € cent) are displayed below the avatar in the moment of reward collection (not visible here)

In value-based decision tasks, choice perseveration can be studied through a sequential manipulation of the combined values of the options (see “Design”), which can be realized by varying either one feature or all feature dimensions of the options. Previous research showed in a nonverbal decision task (Scherbaum et al. 2013, 2018b) that the sequential manipulation of one feature (i.e., distance of an option) produces choice perseveration as predicted by the attractor model (Scherbaum et al. 2016, their Experiment 3; Senftleben et al. 2019a, b). In this nonverbal decision task, participants collect different coins by playing an avatar which they move on a checkered playing field by clicking with the computer mouse (Fig. 3). In each trial, participants have to choose between two options of different reward magnitude (small vs. large) at different distances (near vs. far fields). The playing field stays constant across trials—except the options which change from trial to trial—and the avatar starts each trial from the position of the previously chosen option.

The attractor model for these tasks combines the features of the task’s options, namely reward value and distance, into one combined value and hence assumes that choice perseveration should occur irrespective of whether one manipulates reward values, distances, or both. From this assumption, we derived three hypotheses for our current study. We hypothesized that we would (H1) replicate choice perseveration with a sequential manipulation over the distance between the options,^1^ as well as (H2) find choice perseveration with a sequential manipulation over the reward magnitudes or all features together, that is a combination of distance and reward. Furthermore, we expected that (H3) the perseveration effect would not differ between the different variants of the sequential manipulation. We corroborated the theoretical derivation of our hypotheses by computational simulation of the attractor model (please see osf.io/7fb5q for simulation script and the “Appendix” for a more elaborate explanation of the model).

## Methods

### Participants

We recruited forty-three participants (65% female, mean age = 22.98 years, SD = 5.06 years) through the department’s database system ORSEE (Greiner 2015). The experiment was conducted at the Technische Universität Dresden and was approved by the university’s institutional review board. All had normal or corrected-to-normal vision and gave informed consent prior to data collection. According to experimental protocol, three participants finished the experiment after the first block due to individual choice behavior not allowing for a sufficient sequential manipulation of subjective values in the subsequent blocks. Hence, we archived a final sample size of forty participants (70% female, mean age = 23.05 years, SD = 5.21 years) for all subsequent analyses. Participants received a 2.50 € show-up fee and the money they collected within the experiment (mean = 3.17, SD = 0.39).

### Apparatus and stimulus

The experiment was presented on a 17-inch screen (1280 × 1024 pixels, 85 Hz). As presentation software, we used Psychophysics Toolbox 3 (Brainard 1997; Pelli 1997) in MATLAB 2010b, running on a Windows XP SP2 personal computer. Responses were carried out by moving a computer mouse.

Participants moved an avatar on a field divided into 20 × 20 fields (Fig. 3). Each trial consisted of two options (coins, one small and near—SN—and the other large and far away—LF) positioned in such a way that the first move into one direction decreased the distance to one option but increased the distance to the other option. For both options, a number printed on each coin represented the *reward* and the horizontal and vertical distance to the avatar represented the *distance*. Rewards ranged from 1 to 99 credits and distances ranged from 2 to 15 fields. Trials can also be described in relations: The difference of both distances computes the *interval*; the ratio of both rewards computes the *reward ratio*.

### Procedure

Participants’ task was to choose between two rewards and to collect as much reward as possible within the allotted time limit (4 blocks, 8 min per block, different number of trials per block dependent on the duration of each trial).

A trial started with an inter-trial interval (ITI) of 1.3 s. Within this interval, the mouse cursor was locked on the avatar. After the ITI, the two options were presented and participants could click on the adjacent fields to move the avatar. When the avatar reached one option, both options disappeared, the reward of the selected option was added to the credits, and the next trial started.

Between blocks, participants were informed about their credits and were instructed to rest briefly before the self-paced start of the next block.

Before the start of the experimental blocks, participants worked through a test block of 2 min to get used to the virtual environment, handling of the computer mouse, and the range of rewards and distances.

### Design

The experiment consisted of four blocks: the *assessment bloc*k and three experimental blocks. The initial assessment block differed conceptually from the three experimental blocks as its aim was to measure participants’ individual choice behavior to configure the succeeding experimental blocks. Over the experimental blocks, we realized different sequential manipulations of the options’ subjective values. The sequential arrangement of the experimental blocks was varied and balanced across participants.

In the assessment block, rewards ranged from 11 to 99 and distances from 2 to 15. The ranges were given by orthogonally varying the intervals (1, 4, 8, and 12 fields), the reward ratios (20, 50, 70, 80, 88, 93, 97, and 99%), and the distance of the SN option (2 and 3 fields); the rewards of the LF option were randomly chosen from a discrete uniform distribution between 55 and 99. The combination of 8 reward ratios, 2 distances of the SN option and 4 intervals yielded a complete set of 64 trials. We generated 5 such sets, with a randomized order of trials within each set.

For the sequential manipulation, we described participants’ choice behavior by estimating for each interval the reward ratio at which the subjective values of both options were equally high (i.e., *indifference points*). Based on those estimates, we tailored trials compatible to the respective sequential manipulation. The basic structure of the sequential manipulation is a stepwise change of the options’ subjective values in opposite directions. In our trial sequences, we aimed to change the subjective values in 12 steps as indicated by the differences between the indifference points and the reward ratio of the trials (− 0.3000, − 0.2455, − 0.1909, − 0.1364, − 0.0818, − 0.0273, 0.0273, 0.0818, 0.1364, 0.1909, 0.2455, 0.3000), that is the *manipulation points*.^2^ A negative manipulation point denotes a superior subjective value of the LF option; a positive manipulation point denotes a superior subjective value of the SN option. The superiority of either option positively correlates with the absolute value of manipulation points. Please note that the interpretation of the manipulation point is analog to the interpretation of the control parameter in the computational simulation (see “Appendix” and osf.io/7fb5q).

We then applied this manipulation in three different experimental blocks. Within each block, we varied the direction of the sequential manipulation (*direction* = SN to LF or LF to SN) and created eight sequences for each direction. This resulted in 16 possible sequences, and hence 192 trials.

In the *distance block*, we consecutively increased or decreased the distance of the LF option while keeping all other factors constant within the sequence. For each sequence, the distance of the SN option and the reward of the LF option were randomly chosen from discrete uniform distributions between 2 and 3 fields, and 55 and 99 credits, respectively. The reward of the SN option was randomly drawn from the uniform distribution between participants’ two indifference points at the medium intervals (i.e., [6,7]).

In the *value block*, we consecutively increased or decreased the reward of the SN option while keeping all other factors constant within the sequence. For each sequence, the distance of the SN option and the reward of the LF option were randomly chosen. The distance of the LF option was randomly drawn from the set of intervals at which participants’ indifference points plus or minus the respective manipulation points were valid (0 < *x* < 1). For each trial within the sequence, the rewards of the SN option were then calculated.

In the *combined block*, we consolidated the former manipulations and varied both the distance of the LF option and the reward of the SN option in such a way that the manipulation points consecutively increased or decreased within the sequence. For each sequence, the distance of the SN option and the reward of the LF option were randomly chosen. For each trial within the sequence, the distance of the LF option was randomly chosen from the set of intervals at which the respective manipulation was valid. The rewards of the SN option were then calculated.

## Results

On average, participants completed 134 trials (SD = 23) in the measurement block, and chose the SN option in 56.11% (SD = 18.18) of the trials. The aim of the assessment block was to measure participants’ choice behavior as described by indifference points. Indifference points were given by the point of inflection of a logistic function that was fitted to participants’ choices as a function of increasing reward ratios.^3^

In the three experimental blocks, participants completed 387 trials (SD = 67) on average. Hence, on average, participants ran through 32 sequences (SD = 6), consisting of 16 sequences in each direction (SD = 3, respectively). The SN option was chosen in 48.37% (SD = 22.19) of the trials.

**Fig. 4:**
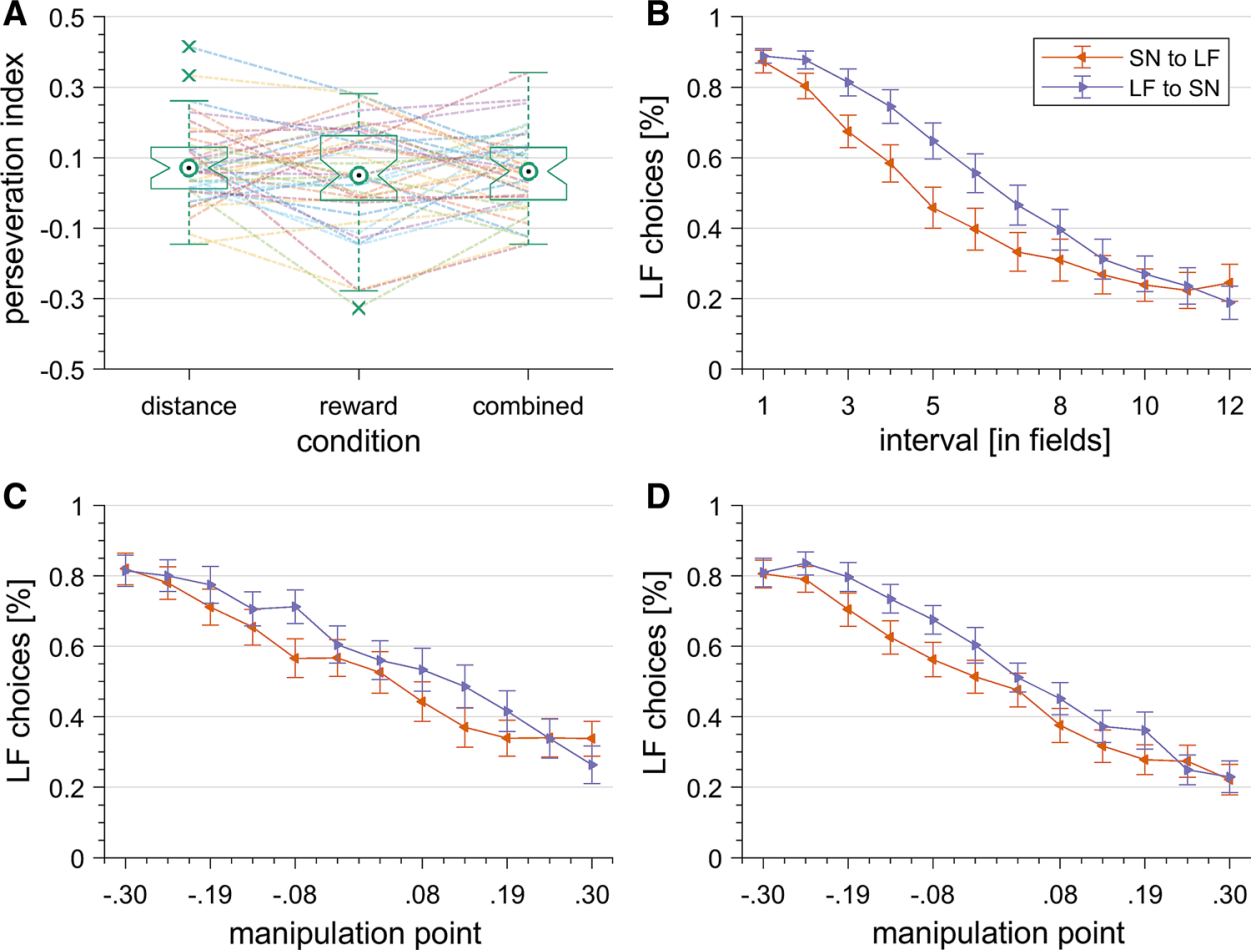
Results of the Experiment. **a** Distributions and box plots of the perseveration index between experimental conditions. **b**, **c**, **d** Average perseveration plots between experimental conditions. Plots depict participants’ mean response pattern (i.e., percentage of LF choices) over intervals (**b**) or manipulation points generated by sequential manipulation over rewards (**c**) or a rewards and distances (**d**). Note: Error bars depict standard error. Colors indicate directions

Our first two hypotheses (H1 and H2) stated that choice perseveration would occur in each experimental condition. Therefore, we summarized choice ratios into one perseveration index by calculating the differences between participants’ choice ratios in sequences of either direction. As expected, separate one-sample *t* tests (> 0) revealed significant choice perseveration in the distance block (*M* = 0.082, SD = 0.11), *t*(39) = 4.61, *p* < .001, *d* = 0.73, BF_10_ = 2410.77, the reward block (*M* = 0.046, SD = 0.15), *t*(39) = 1.93, *p* = .030, *d* = 0.31, BF_10_ = 3.08, and the combined block (*M* = 0.057, SD = 0.11), *t*(39) = 3.16, *p* = .002, *d* = 0.50, BF_10_ = 77.17 (see Fig. 4a).^4^ Although the (frequentist) significance of those results supported our first two hypotheses (H1 and H2), the (Bayesian) evidence in favor of the assumed perseveration effect for the reward block was not overwhelming and ranged from inconclusive to moderate in a sensitivity analysis.^5^ The statistical evaluation on the aggregate level was also reflected qualitatively by the inspection of choice perseveration on a more detailed level as depicted in Fig. 4b–d.

Our third hypothesis (H3) stated that choice perseveration would not differ between experimental conditions. A repeated measures ANOVA on the perseveration index yielded no main effect, *F*(2,78) = 1.22, *p* = .30, *η*^2^ = .03, BF_01_ = 4.6, supporting our hypothesis (see Fig. 4a).

Since our analyses on an aggregate level yielded somewhat borderline results specifically with respect of the perseveration effect in the reward block (H2) as well as the equality of the perseveration effect across all experimental blocks, we also tested our hypotheses with a generalized linear mixed model (GLMM) at the trial level using a logistic link function and the Laplace approximation. The analysis was implemented using *glmer* function of the *lme4* package for R (Bates et al. 2014). For each model, we ran both a version with random intercepts and fixed slopes, and a version with random intercepts and random slopes. We always chose the better fitting model based on the Bayesian information criterion (BIC), which was always the model with random intercepts and random slopes (for more information, please see the respective analysis script at osf.io/7fb5q).

Similar to our analysis on the aggregate level, we tested the effect of direction on choice separately for each experimental block and without collapsing the interval/manipulation point variable (H1 and H2). This GLMM approach also permitted to translate the manipulated intervals in the distance block into individualized manipulation points, which makes the separate analyses even more comparable. Hence, our three separate models included random intercepts and random slopes for each participant as well as fixed main and interaction effects for manipulation point and direction. The results show that direction significantly impacts participants’ choices in all experimental blocks, corroborating our prior analysis and conclusion (see Table 1).

**Table 1:** Parameter of a generalized linear mixed-effect model analyzing choice as a function of manipulation point and direction

Block	Weight	Estimate	Std. error	*Z* value	*Pr*(> |*z*|)
distance	(Intercept)	− 1.14	0.37	− 3.05	< 0.01
	Manipulation point	− 12.18	1.41	− 8.61	< 0.01
	Direction	0.82	0.18	4.59	< 0.01
	Manipulation point by direction	− 0.09	1.16	− 0.08	0.94
Reward	(Intercept)	0.20	0.30	0.66	0.51
	Manipulation point	− 6.07	0.58	− 10.48	< 0.01
	Direction	0.44	0.19	2.25	0.02
	Manipulation point by direction	− 1.04	0.65	− 1.60	0.11
Combined	(Intercept)	− 0.09	0.22	− 0.41	0.68
	Manipulation point	− 6.19	0.47	− 13.22	< 0.01
	Direction	0.39	0.12	3.36	< 0.01
	Manipulation point by direction	− 0.68	0.55	− 1.24	0.21

The model included a random intercept and a random slope for participant. The model was fitted using a logistic link function and the Laplace approximation

In order to test the effect of the experimental condition on choice perseveration (H3), we ran a null model across all experimental conditions including the same random intercept and random slope structure as before and tested it against an alternative model with the same structure but incorporating the experimental condition nested within participants. The results show that the alternative model (BIC = 13,852, LL = − 6810.3; *Χ*^2^(10) = 1204.1, *p* < .001) fitted the data better than the null model (BIC = 14,960, LL = – 7412.3), indicating that the experimental condition indeed induced a significant amount of variation to the choice data (for details, please see the respective analysis script at osf.io/7fb5q). Hence, the result from the GLMM analysis contradicts our prior analysis and conclusion.

## Discussion

In this study, we aimed at testing a simplification of recent models of delay discounting decisions, that is, the summation of different sub-processes into one description of the overall process dynamics. Our results suggest that this simplification seem to be valid in principle: We studied choice perseveration in a nonverbal decision task by sequentially manipulating the subjective values of the options; we found choice perseveration for all three feature dimensions that were used for the manipulation. Hence, we replicated previous findings (Scherbaum et al. 2016; Senftleben et al. 2019a, b), as well as, collected evidence that the simplification introduced by the low-dimensional attractor model seems to be valid with respect to modeling high-level decision-making processes (H2). However, we revealed mixed evidence with regard to the model’s prediction that the strength of choice perseveration would not differ between the feature dimensions used for sequential manipulation (H3).

In the following, we will first discuss the role of the assumption for modeling decision making, before we turn to the question why the manipulation of different feature dimensions might lead to different strengths of the perseveration effect.

While we tested this simplification for a prediction of the attractor model, other computational models of decision making introduce similar simplifications: Basic sequential sampling models such as *drift diffusion* models (e.g., Krajbich et al. 2010) or *linear ballistic accumulator* models (e.g., Rodriguez et al. 2014) also capture the decision process on the level of competing subjective values (for an overview see Ratcliff et al. 2016). These models explain many effects found in empirical data in concordance with their neural underpinnings (Rodriguez et al. 2015) and their process dynamics (Resulaj et al. 2009), which additionally supports the validity of the discussed simplification.

However, we tested the assumption for the attractor model as this model and models with comparable dynamics extend the dynamic process of sequential sampling models: They broaden the short-term focus of sequential sampling models to a perspective that examines nonlinear dynamics of decision making *across multiple decisions*. As those nonlinear dynamics on the long-term timescale naturally arise from attractor models, they yield higher predictive power than basic sequential sampling models which are often seen as a linear simplification of attractor models (Brown and Heathcote 2008; Trueblood et al. 2014).

Consequently, the question arises whether the simplification applied in attractor models and their linear derivatives (as outlined above) implies that all relevant features of the decision task are also represented one-dimensionally? Indeed, there is a substantial body of evidence suggesting that decision making is performed via hierarchical competition processes (Busemeyer and Townsend 1993; Glöckner and Betsch, 2008; Hunt et al. 2014; Roe et al. 2001; Scherbaum et al. 2012; Tsetsos et al. 2010; Usher and McClelland 2004). Such models yield a higher number of hierarchically structured levels, and hence, they model the stream of information processing from the actual input (e.g., distance and reward) to the output (e.g., choice), and competition does not exclusively occur on the option level but also at the feature level (Hunt et al. 2014).

**Fig. 5:**
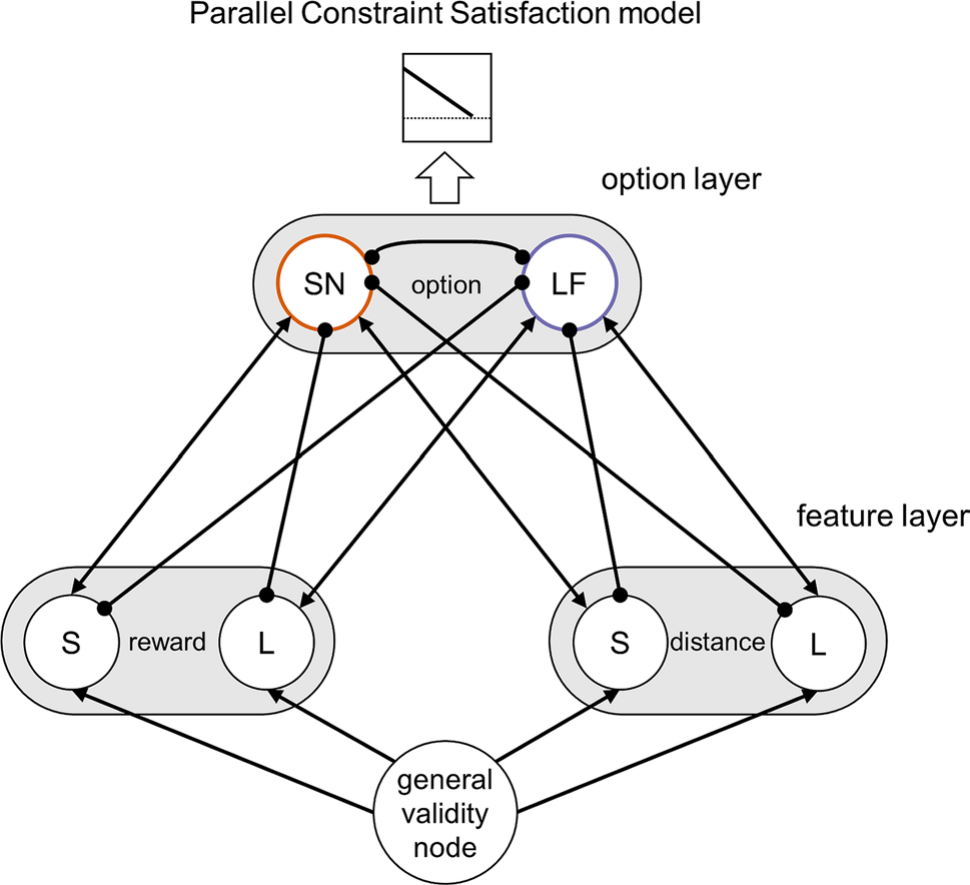
Parallel constraint satisfaction (PCS) model in which the general validity node activates the units of the feature layers. The units of the two feature layers (representing distances and rewards of the options) are connected through inhibitory or excitatory bidirectional connections with each unit of the option layer; the units of the option layer inhibit each other. Response is elicited when the stability of activations in the network reaches a pre-defined threshold (i.e., changes in the weighted sum of the products of all activations fall below this threshold)

Parallel constraint satisfaction (PCS) models (e.g., Glöckner et al. 2014) are one instantiation of such hierarchical models. For our paradigm, an exemplary PCS model would exhibit two levels: the decision level and the feature level, which are highly interconnected (see Fig. 5). This high number of feedback loops between and within levels puts the overall dynamics in narrow bounds, which proposes to summarize them in only a few or even one collective variable constituting the very simplification as applied by attractor models (Kelso 1997). We presented a test case supporting the validity of the simplification when capturing choice dynamics on a long-term timescale; the higher resolution of hierarchical models might be beneficial, though the attractor model also proved to be successful with this respect (Scherbaum et al. 2016, 2018a).

Though we found the perseveration effect for all feature manipulations, our results suggest that the choice dynamics on a long-term timescale might differ across the features (e.g., distance vs. reward). Though this cannot be explained by the attractor model, the exemplary PCS model provides insight in how this difference could come up: In PCS models, the importance of the features is represented by its connection to the general validity node (see Fig. 5). If this connection differed between the distance and the reward representation, then it would be plausible that the short- and long-term dynamics differed as well. Hence, though the one-dimensional attractor dynamics captured the overall process of decision making quite well, such differences in the weighting of the features might yield quantitative, though not qualitative, differences.

Connected to this reasoning, this is a second and even simpler potential explanation for the found differences. Since we do not know the absolute scales of distance ranges and reward ranges, difference could even occur, because the empirical paradigm induces different weights to the features. Hence, both theoretical and methodological considerations provide explanations for the subtle, yet detectable differences between the manipulations of different features.

## Conclusion

In sum, our findings support the validity of attractor models for value-based decision making by understanding its simplification: The system-wide dynamics of the decision system can be summarized collectively on an abstract level without a loss of validity in the description of the decision process (cf. Eckhoff et al. 2011; Wang 2012). Attractor models, hence, not only provide a promising path to understand the interactive dynamics of behavioral phenomena and their neural underpinnings for basic cognitive functions such as memory and perception, but also extend to higher cognitive functions such as value-based decision making. In doing so, our research contributes to the ongoing paradigmatic shift of psychological (decision) science coming from an outcome-based perspective toward a more process-orientated paradigm (Oppenheimer and Kelso 2015; Schulte-Mecklenbeck et al. 2017).

## Figures and Tables

**Fig. 6:**
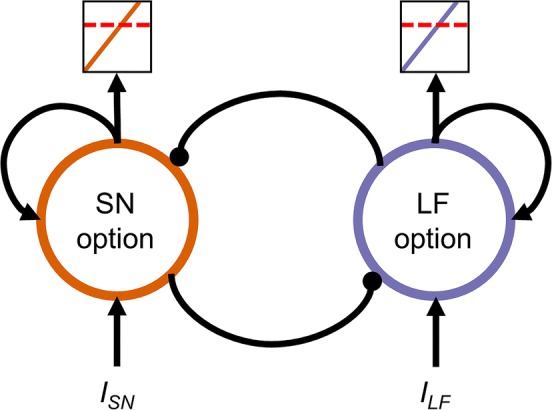
Sketch of the formalized low-level attractor model. Each units’ activation represents the subjective value of one option and inhibits the activation of the alternative option. When an option’s activation reaches the choice-threshold, the choice of this option is elicited

**Fig. 7:**
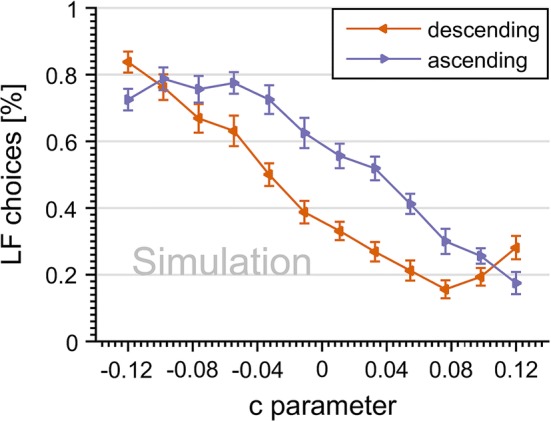
Results of the simulation. Choice perseveration can be summarized as the area between the two lines illustrating the model’s choices over sequences of descending and ascending control parameters

## Appendix

In this computational simulation of the dynamics in value-based decision making, we use a previously published, formalized attractor model (see Scherbaum et al. 2016). Hence, we describe the architecture of the model and the simulation procedure very briefly. Instead, we elaborate on the significant differences between our low-dimensional attractor model and other high-dimensional, dynamic, connectionist models such as parallel constraints satisfaction models (Glöckner and Betsch 2008; Glöckner et al. 2014), decision field theory (Busemeyer and Townsend 1993; Roe et al. 2001), and leaky competing accumulator models (Scherbaum et al. 2012).

## I Model architecture and mathematical description

The model consists of two self-excitatory neural units that are coupled by mutual inhibition (see Fig. 6). Activation of each unit is calculated by separate nonlinear first-order differential equations (Amari 1977; Erlhagen and Schöner 2002; Hock et al. 2003; Noest et al. 2007).

$$ \tau \dot{u}_{\text{SN}} = - u_{\text{SN}} + h + w_{r} \cdot \sigma \left( {u_{\text{SN}} } \right) + w_{i} \cdot \sigma \left( {u_{\text{LF}} } \right) + I_{\text{SN}} + \varepsilon $$

$$ \tau \dot{u}_{\text{LF}} = - u_{\text{LF}} + h + w_{r} \cdot \sigma \left( {u_{\text{LF}} } \right) + w_{i} \cdot \sigma \left( {u_{\text{SN}} } \right) + I_{\text{LF}} + \varepsilon $$

Here, τ denotes the timescale (defining the step size of the Euler solution), *h* the resting level, *w*_*i*_ the (inhibitory) coupling strength of the two units, and *w*_*r*_ the recurrent feedback; *I*_*SN*_ and *I*_*LF*_ define the input into the units representing the subjective value of the two options, ε denotes random noise (*N*[0, 0.1]) and *σ* a sigmoid nonlinearity, mirroring nonlinear neural population dynamics: $\sigma \left( u \right) = \frac{1}{{e^{{ - \beta \left( {u - a} \right)}} }}$. Hence, the nonlinearity of *σ* limits interactions in the network only to the extent that the activation exceeds a soft threshold (Erlhagen and Schöner 2002).

The equation of motion that defines the dynamics of this system used for simulation is derived by differentiation. We simulated the behavior of the derived dynamical system by numerical integration for each trial having a maximum length of 200 time steps; inputs were active after $t_{iti}$ time steps constituting the system’s relaxation time between trials during which the each unit’s activation slowly decays; responses (i.e., choices) were elicited when either unit reaches the activation threshold (see Fig. 6). Results were obtained using MATLAB 2015a running under Windows 10. See Table 2 for a list of the basic parameter in the computational simulation.

**Table 2:** Basic parameters of the model used for simulation

Parameter	Values
$\tau$	10
$h$	− 5
$w_{i}$	− 7
$w_{r}$	6
$I_{{}}$	6
$\varepsilon$	*N*(0,0.1)
$a$	0
$\beta$	*N*(1,0.2)
Time steps	200
*t*_ITI_	55
Threshold	0.85

## Simulation of choice perseveration

In our simulation, we varied only the control parameter *c* that modulates the continuous dynamics of the system (Case et al. 1995; Rączaszek et al. 1999; Scherbaum et al. 2008; Tuller et al. 1994). Here, the control parameter represents the difference of the subjective values between the options and was realized via the strength of the two Inputs *I*_SN_ and *I*_LF_ relative to the general input $I - I_{\text{SN}} = I + \frac{c}{2}$, and $I_{\text{LF}} = I - \frac{c}{2}$. Hence, the lower *c*, the lower the input to the SN option and the higher the input to the LF option, leading to a higher probability of *u*_LF_ winning the competition over *u*_SN_; vice versa for a higher *c*, leading to a higher probability of *u*_SN_ winning the competition over *u*_LF_ (i.e., *c* < 0 favors the LF option; *c* > 0 favors the SN option; *c* = 0 favors no option). However, this relationship only holds when both units are in the same starting state (i.e., an activation $u_{\text{SN}} - u_{\text{LF}} = 0$). In contrast, if the starting state differs (i.e., $u_{\text{SN}} - u_{\text{LF}} \ne 0$), the unit with lower input might win the competition due to its initial advantage that arises from residual activation and causes choice perseveration (Alós-Ferrer et al., 2016; Bonaiuto et al., 2016; Hämmerer et al., 2016).

## Methods

In the simulation, one unit represented the SN and the other unit the LF option (see Fig. 1). We varied the control parameter *c* in 12 steps (− 0.119 − 0.098 − 0.076 − 0.054 − 0.032 − 0.010 0.010 0.032 0.054 0.076 0.098 0.119) and generated trial sequences of 12 trials in which each instantiation of *c* was realized. We also varied the direction of those trial sequences (*direction* = descending [SN to LF] or ascending [LF to SN]) and created eight sequences for each direction. This resulted in 16 possible sequences and hence 192 trials. The order of the trial sequences was randomized.

We simulated 20 participants. The gain parameter β was randomly drawn between participants; random noise ε was realized within participants for each time step; the inputs for both units were activated after fixed *t*_ITI_ = 55 (see Table 2). Please see the modeling script available online for further details and reproducibility (osf.io/7fb5q).

## Results

The simulation showed choice perseveration. We summarized choice ratios into one perseveration index by calculating the differences between mean choices in trial sequences of either direction. A one-sample *t* test (> 0) revealed significant choice perseveration (*M* = 0.11, SD = 0.12), *t*(19) = 4.11, *p* < .001, *d* = 0.92 (Fig. 7), showing that residual activation from the previous decision interacts with the modulating effect of the control parameter on the continuous dynamics of the system in such a way that the system chose the option with the lower subjective value due to an initial advantage from previous decisions (Fig. 2).

## Differences to high-level connectionist models

The model architecture and the mathematical description perfectly illustrates that our low-dimensional attractor mode (as well as its linear derivatives) captures the dynamics of decision making on the subjective value level. Hence, it is invariant to specific values of the options’ features that determine the options’ subjective values, which constitutes a significant simplification. This simplification makes separate simulations for manipulations on the feature level rather than the subjective value level meaningless. Yet, in experimental procedures, the subjective value can be manipulated differently (i.e., via only one or a combination of the options’ features), which we did in the associated study, and therefore tested the validity of the simplification.

In contrast, other high-dimensional, connectionist models comprise more levels of processing including a layer for the options’ features (e.g., Glöckner et al. 2014; Scherbaum et al. 2012). Such models might be sensitive to specific values of the options’ features that determine the options’ subjective values making separate simulation for manipulations on the attribute level eligible.

However, our empirical and simulation data suggest that those separate simulations should lead to similar results irrespective of how the subjective values are manipulated. Hence, our empirical results constitute a new benchmark for testing the validity of high-level, connectionist models that should capture choice perseveration in value-based decision making.

## Footnotes

1 A power analysis (based on recent findings; see Scherbaum et al. 2016, Experiment 3) revealed an approximate sample size of 19–43 participants to detect a medium to large effect with a probability of 95%.

2 For instance, a participant’s indifference point at interval 1 is 0.8. Given a manipulation point of − 0.3, the respective manipulated trial must yield a reward ratio of 0.5 at an interval of 1.

3 The fitting of the logistic regression model was performed using the StixBox mathematical toolbox by Anders Holtsberg (http://www.maths.lth.se/matstat/stixbox/). The fit was based on the model $\log \left[ {\frac{p}{1 - p}} \right] = 1 + Xb$, where *p* is the probability that the choice is 1 (SN) and not 0 (LF), *X* represents reward ratio, and *b* represents the point estimates for the logistic function. .

4 Bayes factors (BF) were calculated with an informed normal-distributed prior assuming a medium effect, *N*(0.5, 0.1).

5 In the sensitivity analysis, we tested the robustness of the BF to different priors. Using uniformed Cauchy priors with various width (i.e., 0–1.5), we found BFs in the reward block ranging from 1.02 to 2.75. We also tested other plausible informed normal priors, *N*(0.5,0.3), *N*(0.5,0.5), *N*(0.3,0.1), *N*(0.3,0.3), *N*(0.3,0.5), revealing BFs in the range from 2.04 to 5.16, and hence, anecdotal to moderate evidence.
